# Online patient feedback: a cross-sectional survey of the attitudes and experiences of United Kingdom health care professionals

**DOI:** 10.1177/1355819619844540

**Published:** 2019-06-02

**Authors:** Helen Atherton, Joanna Fleming, Veronika Williams, John Powell

**Affiliations:** 1 Associate Professor, Warwick Medical School, University of Warwick, UK; 2 Research Fellow, Warwick Medical School, University of Warwick, UK; 3 Senior Researcher, Nuffield Department of Primary Care Health Sciences, University of Oxford, UK; 4 Associate Professor, Nuffield Department of Primary Care Health Sciences, University of Oxford, UK

**Keywords:** consumer behaviour, internet, nurses, physicians, policy

## Abstract

**Objectives:**

Online patient feedback is a growing phenomenon but little is known about health professional attitudes and behaviours in relation to it. We aimed to identify the characteristics, attitudes and self-reported behaviours and experiences of doctors and nurses towards online feedback from their patients or their carers.

**Methods:**

We conducted a cross-sectional self-completed online questionnaire of 1001 registered doctors and 749 nurses and midwives involved in direct patient care in the United Kingdom.

**Results:**

Just over a quarter (27.7% or 277/1001) of doctors and 21% (157/749) of nurses were aware that patients/carers had provided online feedback about an episode of care in which they were involved, and 20.5% (205/1001) of doctors and 11.1% (83/749) of nurses had experienced online feedback about them as an individual practitioner. Feedback on reviews/ratings sites was seen as more useful than social media feedback to help improve services. Both types of feedback were more likely to be seen as useful by nurses compared with doctors and by hospital-based professionals compared with those based in community settings. Doctors were more likely than nurses to believe that online feedback is unrepresentative and generally negative in tone. The majority of respondents had never encouraged patients/carers to leave online feedback.

**Conclusions:**

Despite enthusiasm from health policymakers, many health care professionals have little direct experience of online feedback, and rarely encourage it, as they view it as unrepresentative and with limited value for improving the quality of health services. The difference in opinion between doctors and nurses has the potential to disrupt any use of online patient feedback. The findings have implications for policy and practice in how online patient feedback is solicited and acted upon.

## Introduction

Many sectors now harness online feedback from their customers to drive quality improvement and enhance patient choice, and policymakers have pushed for its inclusion in health care settings, recognizing the potential for growth of this form of feedback in line with increasing use of the internet.^1–3^

Some care organizations have policies and staff dedicated to soliciting and responding to feedback left online^4^ or creating repositories for patients to share their experiences, such as Kaiser Permanente’s ‘Share your story’ facility.^5^ Others solicit feedback from patients on their individual experiences via dedicated sites such as Care Opinion, which operates in the United Kingdom (UK) and Australia, actively inviting patients to share feedback.^6,7^ Community settings have been slower to embrace online patient feedback; in the UK, there has been reliance on comprehensive feedback mechanisms via structured surveys,^8^ despite analysis of the leading UK online feedback site (NHS Choices) showing that the majority of unsolicited comments left by patients relate to experience of care from general practices.^9^

Research has shown that the majority of comments left online by patients are positive in nature^9–11^ and cover similar areas to that obtained via traditional feedback routes.^12^ However, evidence to date suggests that medical professionals, including general practitioners, hospital doctors and surgeons, are wary about online patient feedback, perceiving the content to be largely negative and questioning the representativeness of the patient population.^13–16^ Nurses’ and midwives’ attitudes towards online patient feedback have not previously been reported, despite them being the subject of online patient feedback along with their medical colleagues.^12^

Given that the attitudes held by health care professionals have a major influence on the speed and success of adoption of new technological initiatives in health care settings, there is a need to understand their viewpoint and establish current behaviour in relation to online patient feedback.^17,18^ This information is needed to inform practitioners and policymakers in their decision-making about online patient feedback and its potential for the quality of health care delivered.^19^

We conducted surveys of UK doctors, nurses and midwives with the aim of determining the characteristics, attitudes and self-reported behaviours and experiences of health care professionals in relation to online patient feedback. Our objectives were to measure attitudes, behaviours and experiences and to explore whether these differed by clinician type, professional setting and according to demographic variables including age and gender.

## Methods

### Study design

We conducted a cross-sectional self-completed online questionnaire design. The survey was administered to doctors and nurses/midwives using different routes. This survey is reported in line with the Strobe statement for the reporting of cross-sectional studies.^20^

### Participants

Participants were registered UK doctors, nurses and midwives currently practising in the UK and involved in direct patient care.

### Variables

The survey was designed to identify who uses or has had experience of using online sources of patient feedback and their attitudes towards this type of commentary. We drew on previously conducted research^13,16^ and on policy documents and reports by online feedback organizations^21^ to determine the key elements. The survey comprised eight questions on demographic and professional characteristics and six topic-based questions related to online feedback (see Online Supplement 1, Table S1). Attitudinal questions used Likert-type scales.^22^ The survey questions were piloted in two ways. First, the survey company commissioned to administer the survey to doctors (see section below on data sources) provided guidance and feedback on the survey questions and possible response options based on their extensive experience of surveying doctors on a range of topics. Second, individual local clinicians provided feedback on the wording and order of questions through various iterations of the survey. A lay member of the wider study team also provided feedback on the survey questions at each iteration.

### Data sources

The online survey of doctors was administered via Doctors.net.uk,^23^ a UK online portal and network for the medical profession with 200,000 members. Doctors.net has been previously used in academic surveys of doctors.^24,25^ The survey was administered online via this platform to a quota-sampled^26^ representative group of secondary care (across specialties) and primary care doctors. Doctors received a direct invite via email based on information from their individual doctors.net profile. Doctors were sent the invite until 1000 participants were recruited. The survey was conducted from 12 to 19 July 2016. All participants entering the study were entered into a prize draw.

There was no equivalent route available to survey nurses and midwives. Instead, the same survey questions were included in a planned survey with a wider remit about how nurses and midwives use digital technologies. The online survey link was distributed by the Royal College of Nursing (RCN) via targeted emails sent to RCN nursing forums for e-health, midwifery, district nursing, RCN children and young people’s nursing. It was also distributed via RCN online member bulletins and the RCN twitter feed (@theRCN). In order to bolster the sample, the link to the survey was distributed to 10,000 people registered with the Nursing Times. The survey ran from 17 May to 29 September 2016.

### Study size

The survey of doctors aimed to sample 1000 doctors in total, 500 primary care doctors and 500 secondary care doctors. The survey of nurses aimed to sample at least 500 nurses.

### Quantitative variables and statistical methods

Data from the two survey populations were merged for analysis. Descriptive statistics are presented for variables related to use and chi-squared associations for differences between health care professional groups. We present chi-squared associations between key variables including attitudes and having been subject to online feedback. We used SPSS version 23 for data analysis.

Multivariate logistic regression was used to investigate the way in which different factors were associated with attitudes regarding online comments from patients. Dichotomous variables were created prior to analyses by collapsing the disagree/strongly disagree categories and agree/strongly agree categories, excluding those who neither agreed nor disagreed, or by collapsing ‘never/rarely/sometimes’ and ‘more often than not/all the time’. We conducted analyses on seven different dependent variables relating to attitudes and behaviours. Predictor variables that were considered to be relevant to explain attitudes and behaviours included age, gender, health care professional type (doctor vs. nurse) and setting (community vs. hospital). Community setting included those working in general practice, a hospice, care home or describing themselves as working in the ‘community’. We did not impute missing data. Odds ratios (ORs) and confidence intervals are presented for each independent variable.

For the purposes of presenting the data, we will refer to ‘nurses’ through this category includes midwife participants.

## Results

### Participants and descriptive data

There were a total of 1750 respondents, 1001 were the quota-sampled doctors (n = 501 in primary care; n = 500 in secondary care), and 749 were nurses (n = 715) or midwives (n = 34). The characteristics of respondents are shown below (Table 1).

**Table 1.** Characteristics of participating doctors and nurses.

	Whole sample percentage (n=1750)	Doctors percentage (n=1001)	Nurses and Midwives percentage (n=749)	Overall difference between doctors and nurses/midwives (*P* value)
Gender				
Male	41.0 (717)	64.8 (649)	9.1 (68)	
Female	59.0 (1033)	35.2 (352)	90.9 (681)	<0.001
Age (years)				
Under 30	4.0 (71)	0.9 (9)	8.3 (62)	
30–39	25.5 (446)	33.7 (337)	14.6 (109)	
40–49	31.5 (551)	36.1 (361)	25.4 (190)	<0.001
50–59	32.0 (559)	22.6 (226)	44.5 (333)	
60 or over	7.0 (123)	6.8 (68)	7.3 (55)	
Working hours				
Full-time	70.1 (1227)	74.2 (743)	64.6 (484)	<0.001
Part-time	29.9 (523)	25.8 (258)	35.4 (265)	
Time in practice				
Less than 5 years	6.6 (115)	2.9 (29)	11.5 (86)	
5–10 years	13.4 (234)	17.3 (173)	8.1 (61)	
11–20 years	34.6 (606)	45.0 (450)	20.8 (156)	
21–30 years	24.4 (427)	24.0 (240)	25.0 (187)	
31–40 years	18.2 (319)	10.0 (100)	29.2 (219)	<0.001
More than 40 years	2.8 (49)	0.9 (9)	5.3 (40)	
Setting				
General practice	30.1 (527)	50 (501)	3.5 (26)	0.004
Hospital	51.6 (903)	50 (500)	50.1 (403)	
Community^a^	14.5 (254)	N/A	33.9 (254)	
Other	3.8 (66)	N/A	8.8 (66)	

^a^Community settings included nurses and midwives categorizing themselves as working in the community, in a hospice or in a care home.

There were differences between doctors and nurses, more doctors were males (64.8%) and the majority of nursing respondents were females (90.9%). Most doctors were aged 30 to 49 years (69.8%). For nurses and midwives, the most common age group was 50 to 59 years (44.5%). These proportions are broadly in line with the working population of doctors and nurses in the UK, though the nurses in our sample were slightly older than the general population of nurses (our sample had 51.8% > 50, UK data show that 46% of nurses are > 45).^27^ There were more nurses/midwives (50.1%) working in hospital settings and around a third (33.9%) were working in community settings; this compares with our quota sampled 1:1 split among doctors (501 working in general practice (community) and 500 based in hospitals).

## Experience of receiving feedback

### Feedback on an episode of care

There was a difference between doctors’ and nurses’ experiences of receiving online feedback about an episode of care in which they were involved (*P* = 0.004) (Table 2). Just over a quarter (27.7% (277/1001)) of doctors and 21% (157/749) of nurses said they were aware that patients or carers had provided online feedback on an internet review or ratings site about an episode of care in which they were involved. However, 43.2% (432/1001) of doctors and 49.1% (368/749) of nurses did not know.

**Table 2.** Experiences of receiving online patient/carer feedback.

	Yes	No	I don’t know	Overall difference (doctors vs. nurses), *P* value
Feedback from patient/carer about an episode of care involved in				
Doctors n=1001	27.7% (277)	29.2% (292)	43.2% (432)	0.004
Nurses and midwives, n=749	21.0% (157)	29.9% (224)	49.1% (368)	
Feedback from patient/carer about them as individual practitioner				
Doctors, n=1001	20.5% (205)	37.3% (373)	42.3% (423)	<0.001
Nurses and midwives, n=749	11.1% (83)	37.4% (280)	51.5% (386)	

### Feedback on an individual

One fifth (20.5% (205/1001)) of doctors and 11.1% (83/749) of nurses said they had experienced feedback on an internet review or ratings site about them as an individual practitioner. There was a difference between doctors and nurses (*P* < 0.001) (Table 2). Around half (51.5% (386/749)) of nurses and 42.3% (423/1001) of doctors did not know whether any online patient feedback had ever been left about them as an individual practitioner.

## Attitudes

### Usefulness

When asked to what extent they thought ‘*online patient feedback on experiences of NHS care which is captured on internet reviews and ratings sites is useful to help the NHS improve services*’, only 6% (60/1001) of doctors strongly agreed and 32.8% (328/1001) somewhat agreed. However, 25.3% (253/1001) somewhat disagreed with this statement and 15.6% (156/1001) strongly disagreed.

Views among nurses were more positive: the majority either somewhat (52.5% (393/749)) or strongly agreed (21.1% (158/749)) and the minority somewhat (6.4% (48/749)) or strongly disagreed (2.5% (19/749)). Overall, there was a difference between doctors’ and nurses’ views (*P* = 0.000) (Table 3).

**Table 3.** Online patient feedback on experiences of NHS care is useful to help the NHS improve services.

	Strongly disagree	Somewhat disagree	Neither agree nor disagree	Somewhat agree	Strongly agree	Overall difference (doctors vs. nurses), *P* value
Internet reviews and ratings site						
Doctors, n=1001	15.6% (156)	25.3% (253)	20.4% (204)	32.8% (328)	6% (60)	0.000
Nurses and midwives, n=749	2.5% (19)	6.4% (48)	17.5% (131)	52.5% (393)	21.1% (158)	
Social media						
Doctors, n=1001	26.6% (266)	33.3% (333)	16.2% (162)	21% (210)	3% (30)	0.000
Nurses and midwives, n=749	5.2% (39)	16.7%(125)	25.0% (187)	42.2% (316)	10.9% (82)	

NHS: National Health Service.

The same question was asked in relation to the use of social media. Over half of doctors either somewhat (33.3% (333/1001)) or strongly disagreed (26.6% (266/1001)) that this kind of feedback was useful to help improve NHS services. Conversely, over half of nurses either somewhat (42.2% (316/749)) or strongly agreed (10.9% (82/749)) that this kind of feedback was useful to improve NHS services. Overall, there was a difference between doctors’ and nurses’ views (*P* = 0.000) (Table 3).

When multivariate logistic regression was applied, it showed that doctors were less likely than nurses to agree that ‘*online patient feedback on experiences of NHS care which is captured on internet reviews and ratings sites is useful to help the NHS improve services*’ (OR 0.101, 95% CI 0.070–0.146, *P* = 0.000), and community-based health care professionals were less likely than hospital-based professionals to agree (OR 0.315, 95% CI 0.242–0.410, *P* = 0.000). There was no difference according to age or gender (Online Supplement, Table S1).

The same response pattern was observed with regard to social media, with doctors less likely than nurses to agree it was useful (OR 0.162,95% CI 0.119–0.220, *P* < 0.001), and community-based health care professionals less likely than hospital-based professionals to agree it was useful (OR 0.448,95% CI 0.351–0.572, *P* < 0.001). There was no difference between the groups according to age or gender (Online Supplement, Table S1).

The presence (or absence) of positive attitudes towards the benefit of online patient feedback was not associated with whether a health professional had experienced feedback about an episode of care they were involved in, whether through a review website (*P* = 0.292) or social media (*P* = 0.251). For example, there were similar proportions of health professionals with positive attitudes, regardless of whether they had received patient feedback through a review website (52.5%, 229/434), had not received patient feedback (56.2%, 290/516) or did not know (52.5%, 420/800).

### Representativeness of online patient/carer feedback

Two thirds of doctors thought that online patient/carer feedback was unrepresentative, with 26.2% (262/1001) saying it is very unrepresentative and 40.1% (401/1001) saying it is somewhat representative. Only 1% (10/1001) thought it was very representative and 18.7% (187) thought it was somewhat representative. Views were again different in nurses. Only 4.4% (33/749) thought it was very unrepresentative and 19% (142/749) thought it was somewhat unrepresentative. Although only 2.8% (21/749) thought it was very representative, 44.6% (334/749) thought it was somewhat representative. Overall, there was a difference between doctors’ and nurses’ views (*P* < 0.001) (Table 4).

**Table 4.** Views on representativeness of online patient/carer feedback.

	Very unrepresentative	Somewhat unrepresentative	Neither unrepresentative nor representative	Somewhat representative	Very representative	Overall difference (doctors vs. nurses), *P* value
Doctors, n=1001	26.2% (262)	40.1% (401)	14.1% (141)	18.7% (187)	1% (10)	<0.001
Nurses and midwives, n=749	4.4% (33)	19.0% (142)	29.2% (219)	44.6% (334)	2.8% (21)	

### Nature of content

When asked to what extent they thought ‘*online patient feedback on experiences of NHS care which is captured on internet reviews and ratings sites is generally negative*’ over half of doctors either somewhat (42.0% (420/1001)) or strongly agreed (15.4% (154/1001)), and less than a fifth either somewhat (15.8% (158/1001)) or strongly disagreed (1.6% (16/1001)). The views of nurses were different. Most nurses (44.5%) ‘neither agreed nor disagreed’ that it was negative. A third either somewhat (29.4% (220/749)) or strongly agreed (4.7% (35/749)). Overall, there was a difference between doctors’ and nurses’ views (*P* = 0.000) (Table 5).

**Table 5.** Online patient feedback on experiences of NHS care is generally negative.

	Strongly disagree	Somewhat disagree	Neither agree nor disagree	Somewhat agree	Strongly agree	Overall difference (doctors vs. nurses), *P* value
Internet reviews and ratings site						
Doctors, n=1001	1.6% (16)	15.8% (158)	25.3% (253)	42.0 (420)	15.4 (154)	0.000
Nurses and midwives, n=749	2.5% (19)	19.0% (142)	44.5% (333)	29.4% (220)	4.7% (35)	
Social media						
Doctors, n=1001	1.4% (14)	9.4% (94)	23.8% (238)	45.2% (452)	20.3% (203)	0.000
Nurses and midwives, n=749	2.4% (18)	17.2% (129)	45.5% (341)	28.7% (215)	6.1% (46)	

NHS: National Health Service.

In relation to use of social media, 65.4% of doctors either somewhat or strongly agreed that feedback is generally negative, compared with 10.8% who either somewhat or strongly disagreed with this statement. Again, the views of nurses were different; 45.5% ‘neither agreed nor disagreed’. Overall, there was a difference between doctors’ and nurses’ views (*P* = 0.000) (Table 5).

The multivariate regression showed that doctors were more likely than nurses to agree that *online patient feedback on experiences of NHS care which is captured on internet reviews and ratings sites is generally negative*’. (OR 1.887, 95% CI 1.324–2.689, *P* = <0.0001), and community-based health care professionals were less likely than hospital-based professionals to agree (OR 2.835, 95% CI 2.142–3.753, *P* = <0.0001). There was no difference between groups according to age or gender (Online Supplement 1, Table S2).

For social media, again, doctors were more likely than nurses to agree that ‘*online patient feedback on experiences of NHS care which is captured on social media is generally negative*’ (OR 3.645, 95% CI 2.463–5.394, *P* < 0.001), and community-based health care professionals were more likely than hospital-based professionals to agree (OR 2.450, 95% CI 1.792–3.348, *P* < 0.001). There was no difference between the groups according to age or gender (Online Supplement 1, Table S2).

## Behaviours

The majority of doctors never (43.6% (436/1001)) or rarely (28.3% (283/1001)) encourage their patients/patient's carers to leave feedback on internet reviews and ratings sites. Less than 1 in 10 doctors encourage patients *all the time* (1.8% (18/1001)) or *more often than not* (6.5% (65/1001)). Behaviours were similar in nurses with the majority reporting that they *never* (39.6% (296/749)) or *rarely* (22.9% (171/749)) encourage their patients/patient carers to leave feedback on internet reviews and ratings sites. Only a small proportion of nurses encourage patients *all the time* (5.5% (41/749)) or *more often than not* (10% (75/749)). Overall, there was a difference between doctors’ and nurses’ behaviours (*P* = 0.000) (Table 6).

**Table 6.** Engagement with online patient/carer feedback.

	Never	Rarely	Sometimes	More often than not	All the time	Overall difference (doctors vs. nurses), P value
Encourage patients to leave feedback						
Doctors, n=1001	43.6% (436)	28.3% (283)	19.9% (199)	6.5% (65)	1.8% (18)	0.000
Nurses and midwives, n=748	39.6% (296)	22.9% (171)	22.1% (165)	10.0% (75)	5.5% (41)	
Make a change to practice						
Doctors, n=1001	25.9% (259)	32.5% (325)	33.2% (332)	6.8% (68)	1.6% (16)	0.000
Nurses and midwives, n=748	25.3% (189)	18.2% (136)	29.9% (224)	19.3% (144)	7.4% (55)	

In terms of doctors reporting they had actually made a change to their practice due to any online feedback from internet reviews and ratings (all feedback, not necessarily just feedback directed at them as individuals or their teams), only 1.6% (16/1001) reported doing so all the time and 6.5% (65/1001) more often than not, while 33.2% (332/1001) did sometimes and over half rarely (32.5% 325/1001) or never (25.9% (259/1001)). More nurses made a change to their practice due to feedback from internet reviews and ratings, a quarter reported doing so *more often than not* (19.3% (144/749)) or *all the time* (7.4% (55/749)), while 29.9% (224/749) did *sometimes* and 18.2% (136/749) rarely. A quarter of nurses (25.3% (189/749)) said they never made a change to practice. Overall, there was a difference between doctors’ and nurses’ behaviours (*P* = 0.000) (Table 6).

There was a difference in the relationship between thinking patient views are unrepresentative and making a change to practice due to feedback (*P* = 0.000). Among the 838 health professionals who felt that views are unrepresentative, 787 (93.9%) *never*, *rarely* or *sometimes* made a change to practice (low feedback use), compared with 51 (6.1%) who made a change to practice *all of the time* or *more often than not* (high feedback use). Among the 552 health professionals who felt that views are representative, 364 (65.9%) *never, rarely* or *sometimes* made a change to practice (low feedback use) compared with 188 (34.1%) who made a change to practice *all of the time* or *more often than not* (high feedback use).

The multivariate regression showed that doctors were less likely than nurses to have ‘encouraged patients/carers to leave feedback on internet reviews and ratings sites’ (OR 0.537, 95% CI 0.359–0.803, *P* = 0.002) as were those working in a community setting (OR 0.559, 95% CI 0.405–0.771, *P* = 0.000). There was no difference between the groups according to age or gender. Doctors were less likely to agree that they had ‘*made a change to practice because of feedback from internet reviews and ratings sites*’ (OR 0.328, 0.229–0.470, *P* < 0.001). The same pattern was observed for those working in a community setting (OR 0.550, 0.414–0.730, *P* < 0.001). There was no difference between the groups according to age or gender (Online Supplement 1, Table S3).

## Discussion

### Summary of findings

This study presents the first large-scale UK survey exploring the attitudes and behaviours of health care professionals towards online patient feedback. The majority of doctors felt that the feedback was not representative, in direct contrast with nurses where the majority thought it was representative. All health care professionals felt that formal internet review and ratings sites had more potential to be useful in shaping health services than unstructured feedback in social media. We observed that the majority of doctors or nurses rarely or never encourage their patients or patient's carers to leave feedback on internet reviews and ratings sites and when feedback is received, the majority of doctors do not change their practice, though nurses were more likely to change their practice in response to feedback. Both groups were subject to comment, either on an episode of care or on them as a practitioner, but this was more common amongst doctors than nurses. The majority of participants were unaware as to whether feedback had ever been left about them. We found a difference in attitudes between doctors and nurses, with nurses being more positive than doctors about the potential of online patient feedback for health service improvement. We also observed a difference between hospital-based and community-based health care professionals, with hospital-based staff regarding online feedback more positively.

## Strengths and weaknesses of the study

Our survey of doctors used quota sampling^26^ and an online invitation and the survey of nurses used an opt-in approach to a widely advertised online survey invite. These approaches were taken as there was no nationally representative sampling frame available for approaching these professional groups. This approach to recruiting participants may have introduced bias into the sample, particularly in the sample of nurses where participants were recruited via varied online routes and we do not know how many nurses received the invite. The online format favours those who are more comfortable with using the internet. The results should be viewed in light of this self-selected sample. Despite this, the characteristics of the sample broadly reflect the characteristics of doctors, nurses and midwives in the UK in relation to age and gender.^20^ Nurses and midwives were grouped for the purpose of the analysis. However, only 34 of 749 participants were midwives. This was due to the criteria for the survey, which did not exclude midwives but did not target them directly in the recruitment strategy.

The topic of online patient feedback is new and we developed the topic-based questions in the survey ourselves. It is best to use validated questions when conducting a survey, but in the absence of these, we based our questions on existing surveys, obtained input from the survey company administering the doctors survey, and conducted piloting of the survey.

As an observational study, we are identifying associations rather than causation, and these may be indirect due to a common factor unaccounted for in the current analysis. Furthermore, this study was carried out using online surveys and those participants who chose to take part may differ to those who did not, in ways that are not possible to ascertain using our data. They may, for example, be more comfortable with online technologies, especially perhaps in the nurses’ survey, which was specifically presented to potential participants as being about ‘nurses and technology’. Any self-reported measure is subject to potential bias, particularly those questions relating to behaviour that may lead to social desirability bias; however, the anonymity of the survey may have reduced this.

The study findings are specific to doctors and nurses working in the UK and as such are likely to be specific to the context of the NHS and not necessarily generalizable outside of this setting. This is important as online feedback may be deemed to have no influence in driving competition in a nationalized model of health care, compared with other health systems or other sectors where competition for patients exists. Further examination of these relationships in countries with different health systems and among other health professional groups would be beneficial. However, findings from the current study offer important insights towards using online feedback among this group.

## Comparison with other studies

Difficulty in determining how health care professionals might optimize online feedback does not seem to be limited to feedback left online. More broadly, concerns about online feedback identified in this study reflect wider concerns about the provision and collection of patient feedback in general.^28^ UK-based work on the collection of patient experience data in primary care found that staff were sceptical about the value of paper-based patient surveys, their credibility and their ability to support service reconfiguration and quality improvement.^29–31^

It is particularly evident that health care professionals in community settings may require more convincing than their speciality-based colleagues that there are potential positive uses for online feedback. Mirroring our own findings, a survey in Germany found that physicians reporting they had taken measures to improve patient care because of online ratings were more likely to be specialists (57.79%, 946/1637) than general practitioners (50.1%, 207/413) or other providers (44.2%, 137/310, *P* < .001).^11^ Linked work, also in Germany, explored the use of responses to online feedback by physicians, finding that just 1.58% (16,640/1,052,347) of comments on a patient review website had received a response from a physician.

We have conducted a parallel survey of the public, assessing their attitudes and use of online feedback in health care. In that work, we found that many patients are now using online feedback and the main motivations to provide feedback were to inform other patients, to improve standards of NHS services and to praise a service.^11^ This is in contrast to the attitudes of many health professionals in the present study that online feedback is generally negative in content. Our survey of the public did confirm the belief that the people who provide feedback are not representative of the general population. Previous studies with patients have shown that patient and health care professional attitudes may not be aligned and this has implications for the implementation and use of online patient feedback systems.^32^ The adoption and success of innovations such as digital tools are usually dependent on the attitudes and behaviour of those most affected by them.^33^

## Implications for clinicians and policymakers

Current policy promotes the increasing use of online feedback channels for patients, but there is very little evidence base to guide implementation and use, or to inform the training of health professionals in how they might best identify and deal with this feedback. There may be implications from a medico-legal perspective when patients leave information about an individual, and due to medical confidentiality, the health care professional cannot respond directly. It is perhaps understandable that doctors are wary about engaging with a feedback route that has not been fully considered and indicates a need for clear guidance on engagement with this feedback.

There is a key challenge in identifying how best health care professionals might usefully optimize online patient feedback and this differs between groups; the difference in attitudes and behaviours between doctors and nurses and between care settings indicates that different strategies may be needed in different settings.

## Future research

Health care professionals are not widely using feedback to make changes to practice and there is a need to identify how online patient feedback might usefully drive quality improvement, so that health care professionals can understand its value. Further investigation may take the form of observational or experimental studies such as trials of different approaches with outcomes such as care quality metrics. Related work should consider how best to engage health professionals given their limited experience of feedback and their reservations about it.

## Conclusion

Many doctors and nurses in the UK view online feedback from patients as unrepresentative and with limited value for improving health services, especially that derived from social media. Doctors had more negative attitudes towards online feedback compared with nurses, as did community-based health care professionals compared with those working in hospital care, and this has implications for how this feedback is solicited and utilized. We identified a very low proportion of professionals who encourage patients to leave feedback, and this may have implications for the successful introduction of feedback systems, especially if these do not engage with frontline staff regarding how such feedback systems are to be promoted and integrated into everyday health service delivery.

## Supplemental Material

Supplemental material for Online patient feedback: a cross-sectional survey of the attitudes and experiences of United Kingdom health care professionals Click here for additional data file. Supplemental Material for Online patient feedback: a cross-sectional survey of the attitudes and experiences of United Kingdom health care professionals by Helen Atherton, Joanna Fleming, Veronika Williams and John Powell in Journal of Health Services Research & Policy
