# Supplementary material for: Online patient feedback: a cross-sectional survey of the attitudes and experiences of United Kingdom health care professionals
Source: J Health Serv Res Policy. 2019 Jun 2;24(4):235–44. doi: 10.1177/1355819619844540 (PMC7307411; doi:10.1177/1355819619844540)
Supplement: Supplemental material for Online patient feedback: a cross-sectional survey of the attitudes and experiences of United Kingdom health care professionals [file Supplemental_Material.pdf]

**Supplement 1**

**Survey questions**

**Q1 Positive rating of online patient feedback**

In the grid below, please indicate your level of agreement with each statement.

*Please select one response per statement*

| | Strongly disagree | Somewhat disagree | Neither agree nor disagree | Somewhat agree | Strongly agree |
|---|---|---|---|---|---|
| Online patient feedback on experiences of NHS care which is captured on internet reviews and ratings sites is **useful to help the NHS improve services** | ❍ | ❍ | ❍ | ❍ | ❍ |
| Online patient feedback in social media (such as in Tweets on Twitter or in posts on Facebook or a discussion forum like Mumsnet) is **useful to help the NHS improve services** | ❍ | ❍ | ❍ | ❍ | ❍ |

**Q2 Negative rating of online patient feedback**

In the grid below, please indicate your level of agreement with each statement.

*Please select one response per statement*

To what extent do you agree with the following statements?

| | Strongly disagree | Somewhat disagree | Neither agree nor disagree | Somewhat agree | Strongly agree |
|---|---|---|---|---|---|
| Online patient feedback on experiences of NHS care which is captured on internet reviews and ratings sites is **generally negative** | ❍ | ❍ | ❍ | ❍ | ❍ |
| Online patient feedback in social media (such as in Tweets on Twitter or in posts on Facebook or a discussion forum like Mumsnet) is **generally negative** | ❍ | ❍ | ❍ | ❍ | ❍ |

**Q3 Usefulness of online patient feedback**

In the grid below, please rate the frequency that applies to each statement.

*Please select one response per statement*

| | Never | Rarely | Sometimes | More often than not | All the time |
|---|---|---|---|---|---|
| You encourage your patients/their carers to leave feedback on internet reviews and ratings sites? | ❍ | ❍ | ❍ | ❍ | ❍ |
| Your organisation feedback internet reviews and comments left by patients/carers to you or your team? | ❍ | ❍ | ❍ | ❍ | ❍ |
| You make a change to your practice because of feedback from internet reviews and ratings sites? | ❍ | ❍ | ❍ | ❍ | ❍ |

**Q4 Representation of patient views**

How representative of patient views do you think online patient/carer feedback is?

*Please select one option*

Very unrepresentative (1)

Somewhat unrepresentative (2)

Neither unrepresentative nor representative (3)

Somewhat representative (4)

Very representative (5)

**Q5 Ever left online feedback about care**

Have patients/carers ever left online patient feedback on an internet review or ratings site about an episode of care in which you were involved?

Yes (1)

No (2)

I don't know (3)

**Q6 Ever left online feedback about you**

Have patients/carers ever left online patient feedback on an internet review or ratings site about you as an individual practitioner?

| | |
|---|---|
| Yes | (1) |
| No | (2) |
| I don't know | (3) |

**Table S1**

**Online patient feedback on experiences of NHS care is useful to help the NHS improve services**

| | **Internet reviews and ratings** | | | **Social media** | | |
|---|---|---|---|---|---|---|
| **Predictor variables** | **OR** | **95% CI** | **P value** | **OR** | **95% CI** | **P value** |
| Health professional type (doctor vs nurse*) | 0.101 | 0.070-0.146 | 0.000 | 0.162 | 0.119-0.220 | <0.001 |
| Health professional setting (community vs hospital*) | 0.315 | 0.242-0.410 | 0.000 | 0.448 | 0.351-0.572 | <0.001 |
| Gender (male vs female*) | 1.057 | 0.783-1.426 | 0.718 | 1.018 | 0.760-1.364 | 0.904 |
| Age | | | | | | |
| • Under 30 | 1.049 | 0.341-3.232 | 0.933 | 1.112 | 0.507-2.440 | 0.791 |
| • 30-39 | 0.691 | 0.390-1.223 | 0.205 | 1.042 | 0.617-1.758 | 0.879 |
| • 40-49 | 0.770 | 0.439-1.350 | 0.362 | 1.041 | 0.624-1.738 | 0.877 |
| • 50-59 | 0.635 | 0.360-1.121 | 0.117 | 0.988 | 0.591-1.653 | 0.964 |
| • 60+* | | | | | | |

*Reference category

**Table S2:**

**Online patient feedback on experiences of NHS care is generally negative doctors and nurses**

| | Internet reviews and ratings | | | Social media | | |
|---|---|---|---|---|---|---|
| **Predictor variables** | **OR** | **95% CI** | **P** | **OR** | **95% CI** | **P** |
| Health professional type (doctor vs nurse*) | 1.887 | 1.324-2.689 | 0.000 | 3.645 | 2.463-5.394 | <0.001 |
| Health professional setting (community vs hospital*) | 2.835 | 2.142-3.753 | 0.000 | 2.450 | 1.792-3.348 | <0.001 |
| Gender (male vs female*) | 1.040 | 0.742-1.459 | 0.819 | 0.881 | 0.598-1.300 | 0.525 |
| Age | | | | | | |
| • Under 30 | 1.263 | 0.565-2.824 | 0.570 | 1.225 | 0.520-2.885 | 0.643 |
| • 30-39 | 1.568 | 0.873-2.815 | 0.132 | 1.364 | 0.720-2.583 | 0.341 |
| • 40-49 | 1.457 | 0.821-2.588 | 0.199 | 1.548 | 0.826-2.902 | 0.173 |
| • 50-59 | 1.513 | 0.848-2.699 | 0.161 | 1.574 | 0.837-2.958 | 0.159 |
| • 60+* | | | | | | |

*Reference category

**Table S3:**

**Behaviours in relation to online feedback on internet reviews and ratings sites.**

| | **Encouraged patients/carers to leave feedback** | | | **Made a change to practice** | | |
|---|---|---|---|---|---|---|
| **Predictor variables** | **OR** | **95% CI** | **P** | **OR** | **95% CI** | **P** |
| Health professional type (doctor vs nurse*) | 0.537 | 0.359-0.803 | 0.002 | 0.328 | 0.229-0.470 | <0.001 |
| Health professional setting (community vs hospital*) | 0.559 | 0.405-0.771 | 0.000 | 0.550 | 0.414-0.730 | <0.001 |
| Gender (male vs female*) | 1.073 | 0.719-1.601 | 0.731 | 0.837 | 0.578-1.211 | 0.345 |
| Age | | | | | | |
| • Under 30 | 0.957 | 0.407-20252 | 0.921 | 1.125 | 0.532-2.377 | 0.758 |
| • 30-39 | 0.745 | 0.384-1.445 | 0.384 | 0.811 | 0.442-1.488 | 0.499 |
| • 40-49 | 0.900 | 0.480-1.688 | 0.742 | 0.917 | 0.514-1.636 | 0.770 |
| • 50-59 | 1.032 | 0.556-1.916 | 0.921 | 1.283 | 0.733-2.248 | 0.383 |
| • 60+* | | | | | | |

*Reference category
